# Association Between Hypothalamic Volume and Metabolism, Cognition, and Behavior in Patients With Amyotrophic Lateral Sclerosis

**DOI:** 10.1212/WNL.0000000000209603

**Published:** 2024-06-14

**Authors:** Annebelle Michielsen, Kevin van Veenhuijzen, Mark R. Janse van Mantgem, Michael A. van Es, Jan H. Veldink, Ruben P.A. van Eijk, Leonard H. van den Berg, Henk-Jan Westeneng

**Affiliations:** From the Department of Neurology (A.M., K.V.V., M.R.J.V.M., M.A.V.E., J.H.V., R.P.A.V.E., L.H.V.D.B., H.-J.W.), UMC Utrecht Brain Center, and Biostatistics & Research Support (R.P.A.V.E.), Julius Centre for Health Sciences and Primary Care, University Medical Centre Utrecht, the Netherlands.

## Abstract

**Background and Objectives:**

Dysfunction of energy metabolism, cognition, and behavior are important nonmotor symptoms of amyotrophic lateral sclerosis (ALS), negatively affecting survival and quality of life, but poorly understood. Neuroimaging is ideally suited to studying nonmotor neurodegeneration in ALS, but few studies have focused on the hypothalamus, a key region for regulating energy homeostasis, cognition, and behavior. We evaluated, therefore, hypothalamic neurodegeneration in ALS and explored the relationship between hypothalamic volumes and dysregulation of energy metabolism, cognitive and behavioral changes, disease progression, and survival.

**Methods:**

Patients with ALS and population-based controls were included for this cross-sectional and longitudinal MRI study. The hypothalamus was segmented into 5 subregions and their volumes were calculated. Linear (mixed) models, adjusted for age, sex, and total intracranial volume, were used to compare hypothalamic volumes between groups and to analyze associations with metabolism, cognition, behavior, and disease progression. Cox proportional hazard models were used to investigate the relationship of hypothalamic volumes with survival. Permutation-based corrections for multiple hypothesis testing were applied to all analyses to control the family-wise error rate.

**Results:**

Data were available for 564 patients with ALS and 356 controls. The volume of the anterior superior subregion of the hypothalamus was smaller in patients with ALS than in controls (β = −0.70 [−1.15 to −0.25], *p* = 0.013). Weight loss, memory impairments, and behavioral disinhibition were associated with a smaller posterior hypothalamus (β = −4.79 [−8.39 to −2.49], *p* = 0.001, β = −10.14 [−15.88 to −4.39], *p* = 0.004, and β = −12.09 [−18.83 to −5.35], *p* = 0.003, respectively). Furthermore, the volume of this subregion decreased faster over time in patients than in controls (β = −0.25 [0.42 to −0.09], *p* = 0.013), and a smaller volume of this structure was correlated with shorter survival (hazard ratio = 0.36 [0.21–0.61], *p* = 0.029).

**Discussion:**

We obtained evidence for hypothalamic involvement in ALS, specifically marked by atrophy of the anterior superior subregion. Moreover, we found that atrophy of the posterior hypothalamus was associated with weight loss, memory dysfunction, behavioral disinhibition, and survival, and that this subregion deteriorated faster in patients with ALS than in controls. These findings improve our understanding of nonmotor involvement in ALS and could contribute to the identification of new treatment targets for this devastating disease.

## Introduction

Amyotrophic lateral sclerosis (ALS) is a neurodegenerative disease that results in progressive muscle weakness and spasticity, eventually leading to respiratory failure and death.^1^ Owing to neurodegeneration outside the motor system, multiple nonmotor symptoms can occur, such as dysregulation of energy metabolism and impairment of cognition, behavior, and sleep.^2,3^ Both dysregulation of energy homeostasis, and cognitive and behavioral dysfunction have a negative effect on survival and reduce quality of life in patients with ALS.^4-7^ Therefore, investigating extramotor neurodegeneration that underlies metabolic, cognitive, and behavioral impairments gives us greater insight into the extent of nonmotor involvement in ALS and, as potential therapeutic targets, could assist in improving prognosis and quality of life.

The hypothalamus controls fundamental physiologic functions such as energy homeostasis, cognition, behavior, thermoregulation, and circadian rhythms.^8^ It regulates appetite and body weight by activating the melanocortin system in response to peripheral signals, such as insulin, leptin, and ghrelin.^9,10^ Furthermore, as part of the limbic system with interconnections to the hippocampus, amygdala, and prefrontal cortex, the hypothalamus is involved in learning, memory, emotional processing, and motivated and social behavior.^11-13^ Neuroimaging techniques are widely used to investigate nonmotor neurodegeneration in ALS,^14^ but few studies have focused on the hypothalamus.^3^ Existing literature on hypothalamic volumetry in ALS is summarized in eTable 1. These studies are limited by mixed results, with some reporting global atrophy of the hypothalamus while others do not. Because longitudinal research is lacking, it is not known whether hypothalamic neurodegeneration in ALS is progressive over time. Furthermore, evidence for subregional changes and the relationship with metabolic dysfunction, impaired cognitive and behavioral domains, and disease progression are inconclusive.^15-18^ Hence, there is a need for clarity regarding hypothalamic involvement in ALS and an understanding of its role in energy dysregulation, cognitive and behavioral dysfunction, and disease progression.

The purpose of this magnetic resonance study is, therefore, to evaluate the effect of ALS on hypothalamic volumes, both cross-sectionally and longitudinally, in a large data set. Second, we aim to investigate metabolic, cognitive and behavioral changes, disease progression, and survival in relation to the hypothalamus in ALS.

## Methods

### Study Design and Participants

This cross-sectional and longitudinal case-control study included patients with ALS, population-based controls, and familial controls (e.g., asymptomatic family members of ALS patients with a chromosome 9 open reading frame 72 [*C9orf72*] repeat expansion). Participants were recruited between January 2009 and May 2022 from an ongoing population-based study in the Netherlands.^19^ Inclusion criteria were patients aged between 18 and 80 years, no breathing difficulty or risk of suffocation when lying supine, no structural brain abnormality, and no history of stroke or epilepsy. Familial controls were included if asymptomatic (no signs of upper/lower motor neuron disease, bulbar dysfunction, or cognitive or behavioral changes for at least 1 year after MRI). Patients with ALS underwent follow-up scans at 3- to 6-month intervals with a maximum of 5 scans. Controls received follow-up scans with an interval of 1 year after the first scan and then at 3- to 5-year intervals, also with a maximum of 5 scans. *C9orf72* repeat expansion, the most common genetic cause of ALS, was tested in patients and familial controls, as described previously.^20^ A repeat length of ≥30 repeats was defined as pathogenic (*C9orf72*-positive).

### Clinical Assessment

At diagnosis and follow-up, demographic data, disease onset site, bulbar symptoms, frontotemporal dementia (FTD) presence, and survival status were collected. Survival was defined as time between symptom onset and noninvasive ventilation for more than 23 hours per day, tracheostomy, or death from any cause. Symptom duration was measured from first symptom onset to MRI date. Daily functioning, disease progression rate, and presence of dysphagia were determined using the revised ALS Functional Rating Scale (ALS-FRS-R).^21^ Dysphagia was evaluated using question 3 of the ALS-FRS-R (about swallowing), with lower scores indicating more severe swallowing difficulties and higher scores indicating better swallowing ability. Disease progression rate was calculated using the formula (48 − total ALS-FRS-R score)/symptom duration (in months). To assess presence of upper motor neuron or lower motor neuron signs, a neurologic examination was performed at each visit and scored according to the Devine scoring method.^22-24^

### Metabolic Assessment

Weight loss, assessed at diagnosis, was considered present if there was a decrease of more than 2 kilograms body weight. This threshold was selected to distinguish actual weight loss from natural fluctuations in weight. Weight loss was dichotomized because precise information about the number of kilograms lost was unavailable in many patients. Data on weight and height, from which body mass index (BMI) was derived, were collected using a questionnaire within 3 months of diagnosis.^25^ Metabolic assessment performed in a subgroup of 39 patients resulted in data on body composition and energy expenditure. Fat mass and fat-free mass (FFM) were determined by total body plethysmography, using a BodPod system, and used to calculate the predicted resting energy expenditure (pREE) with the Structure 4 equation of prediction equation, according to Nelson.^26^ Energy expenditure was measured by indirect calorimetry using Quark RMR respirometer.^27^ To determine the metabolic index, measured resting energy expenditure (mREE) was compared with the pREE (mREE/pREE × 100%).

### Cognitive and Behavioral Assessment

Patients with ALS and familial controls were cognitively and behaviorally screened using the Edinburgh Cognitive and Behavioural ALS Screen (ECAS) and the ALS-FTD Questionnaire (ALS-FTD-Q) within 3 months of the MRI scan. The 5 cognitive domain scores (language, fluency, executive, memory, and visuospatial) were characterized as normal or abnormal based on Dutch normative data.^28^ Whether behavioral change was present or not was determined for each behavioral domain (behavioral disinhibition, apathy, loss of sympathy/empathy, perseverative/stereotyped behavior, and hyperorality/altered eating behavior) based on the carer interview. According to the revised Strong criteria, cognitive and/or behavioral impairments were determined in patients (ALS with cognitive and behavioral impairment, ALS with cognitive impairment, and ALS with behavioral impairment, respectively).^4^

### MRI Acquisition

All participants underwent structural brain MRI using a 3 Tesla Philips Achieva Medical scanner. A high-resolution T1 weighted image was acquired with the following parameters: 3D fast field echo using parallel imaging; repetition time/echo time = 10/4.6 milliseconds, flip-angle 8°, slice orientation: sagittal, 0.75 × 0.75 × 0.8 mm voxel size, field of view = 160 × 240 × 240 mm, reconstruction matrix = 200 × 320 × 320, covering the whole brain. During follow-up visits, the same imaging protocol was used.

### Processing and Segmentation of the Hypothalamus

The hypothalamus was processed and automatically segmented using the subcortical processing stream available in FreeSurfer version 7.2.^29,30^ Longitudinal processing and segmentation was enhanced by creating an unbiased within-subject template space using robust inverse consistent registration.^31^ Participants with only 1 scan were also preprocessed using the longitudinal preprocessing stream of FreeSurfer. Because the function of hypothalamic lateralization is unclear, we averaged left and right volumes, resulting in a total hypothalamic volume, anterior superior subregion, anterior inferior subregion, superior tubular subregion, inferior tubular subregion, and posterior subregion volume.^32^ We performed manual checks and removed faulty segmentations.

### Statistical Analyses

All analyses were executed using R (version 4.2.2). Clinical and demographic variables were compared using a Mann-Whitney *U* test for continuous variables and Fisher exact test for categorical variables.

### Group Differences in Hypothalamic Volumes

To compare hypothalamic volumes between groups at baseline, linear regression models were used, with total hypothalamic volume, or 1 of the 5 hypothalamic subregion volumes, as outcome variable, and age, sex, and estimated total intracranial volume (eTIV) as covariates. For the between-group comparisons, a grouping variable (e.g., patients with ALS vs controls, ALS *C9orf72*-positive vs ALS *C9orf72*-negative, and familial controls *C9orf72*-positive vs familial controls *C9orf72*-negative) was included as an additional covariate. For comparisons between familial controls, a kinship matrix was added as random effect to address genetic dependencies between participants.

### Correlations With Metabolic, Cognitive, Behavioral, and Clinical Outcomes

To analyze associations between hypothalamic volumes and metabolism, cognition, behavior, and clinical parameters, linear regression models were used with the following variables as covariates in separate analyses: weight loss, FFM, mREE, metabolic index, impaired cognitive domain scores, presence of behavioral change across domains, ALS-FTD-Q total score (log-transformed), ALS-FRS-R, disease progression rate, and symptom duration. To assess the effect of ALS on the relationship between hypothalamic volumes and BMI, a main effect for group (e.g., ALS vs control) and BMI, along with an interaction term between these factors, were added.

### Correlations With Survival

Univariable and multivariable Cox proportional hazard models were used to investigate the relationship between hypothalamic volumes and survival since first MRI scan. For survival analyses, the unit of volume was rescaled to 0.1 cm^3^ (i.e., divided by 100). In the initial univariable model, volumes of hypothalamic subregions were used as a predictor. In addition, because weight loss and behavioral changes are predictors of survival in ALS, patients were stratified based on the presence or absence of these factors, and separate univariable survival analyses were conducted within each subgroup. For the first multivariable model, the linear predictor of the European Network for the Cure of ALS (ENCALS) survival prediction model was included. This predictor is a weighted sum score of independent predictors for survival, as previously described.^33^ Missing data in the predictors of the ENCALS survival model were addressed through multiple imputation, followed by fitting of Cox proportional hazards models and pooling of results using Rubin's rules. In a second multivariable model, we examined the relationship between hypothalamic volumes and survival while adjusting for weight loss and behavioral changes by including them as covariables.

### Longitudinal Assessment of Hypothalamic Volumes

To investigate hypothalamic volume changes over time, all available scans were analyzed using linear mixed-effects models. These models included fixed effects for time (in months), age, sex, and eTIV. Random intercepts and slopes for time per subject addressed between-subject variation. With an interaction term between time and groups (e.g., ALS vs control, weight loss vs no weight loss), we determined whether hypothalamic volume decline differed among groups.

### Corrections for Multiple Hypothesis Testing

Permutation-based corrections with 10,000 permutations were used to correct for multiple hypotheses testing and corrected *p* values <0.05 were considered to reflect a statistically significant effect. Cox proportional hazard model results were corrected for multiple testing using Bonferroni corrections. No correction for multiple hypothesis testing was applied to subgroup analyses with extensive metabolic assessments because of the small sample sizes.

Sensitivity analyses, standard protocol approvals, registrations, and patient consents and data availability are available in eMethods.

## Results

### Participant Demographics and Clinical Characteristics

In total, 564 patients with ALS, 356 controls, and 137 familial controls participated in this study. The demographic and clinical characteristics of the patients and controls are summarized in Table 1. The demographic and clinical characteristics of the familial controls are provided in eTable 2. The cohort of patients with ALS in this study is representative of the general ALS population in the Netherlands.^34^ However, because severely affected patients cannot undergo an MRI, the patients included in our study comprise a relatively mild population compared with the general ALS population in the Netherlands. A comparison between patients included in the study and those not included (e.g., the population-based cohort from which patients were selected) revealed several differences at diagnosis (eTable 3). The median age at diagnosis of the patients included was 5 years younger, the occurrence of bulbar symptoms was 15% lower, and they had a lower incidence of FTD than those not included. Although ALS-FRS-R scores were comparable between the 2 groups, the included patients exhibited a lower ∆FRS and longer survival. Symptom duration was similar in the 2 groups. Besides a lower BMI in the patients with ALS, there were no significant differences in parameters between patients with ALS and controls. A total of 44 patients with ALS and 38 familial controls tested positive for the *C9orf72* repeat expansion.

**Table 1:** Descriptive Characteristics of the Study Groups

	ALS	Control	Missing data, %
N	564	356	
Male	378 (67)	243 (68)	0
Age at first MRI, y	62 (54–68)	63 (55–69)	0
Bulbar symptoms at diagnosis	186 (33)		7
*C9orf72* repeat expansion	44 (8)		11
BMI, kg/m^2^	24 (23–27)^a^	26 (24–28)^a^	9
Weight loss	177 (43)		28
FTD	17 (3)		25
ALS-ci/ALS-bi/ALS-cbi	48 (9)/73 (13)/24 (4)		43
ECAS total score impaired	39 (12)		43
ECAS specific score impaired	39 (12)		43
ECAS nonspecific score impaired	18 (6)		43
ECAS executive domain impaired	34 (6)		43
ECAS language domain impaired	37 (7)		43
ECAS fluency domain impaired	28 (5)		43
ECAS visuospatial domain impaired	28 (5)		43
ECAS memory domain impaired	31 (6)		43
ECAS apathy domain impaired	44 (8)		56
ECAS sympathy domain impaired	32 (6)		56
ECAS disinhibition domain impaired	22 (4)		56
ECAS perseveration domain impaired	28 (5)		56
ECAS hyperorality domain impaired	28 (5)		56
ECAS total behavior domains impaired (1/2/3/4/5)	165 (29)/39 (7)/26 (5)/16 (3)/3 (1)		56
ALS-FTD-Q behavioral change (no/mild/severe)	248 (44)/19 (3)/27 (5)		48
ALS-FTD-Q total score	7 (2–18)		48
No. of scans (2/3/4/5)	300/159/103/66	194/14/1/0	
ALS-FRS-R score	40 (36–43)		3
∆FRS,^b^ points per month	0.50 (0.28–0.81)		3
Symptom duration,^c^ mo	15 (10–24)		0
Median survival since onset, mo	41 (27–71)		0

Abbreviations: ALS = amyotrophic lateral sclerosis; ALS-bi = ALS with behavioral impairment; ALS-cbi = ALS with cognitive and behavioral impairment; ALS-ci = ALS with cognitive impairment; ALS-FRS-R = revised ALS Functional Rating Scale; ALS-FTD-Q = ALS-FTD Questionnaire; BMI = body mass index; ECAS = Edinburgh Cognitive and Behavioural ALS Screen; FTD = frontotemporal dementia.

Data are count (%) or median (IQR), unless otherwise specified.

a Significantly different with *p* < 0.05.

b ∆FRS was calculated as (48 − total ALS-FRS-R score)/(symptom duration in months).

c Symptom duration was calculated as time between symptom onset and date of MRI.

### Group Differences in Hypothalamic Volumes

**Figure 1:**
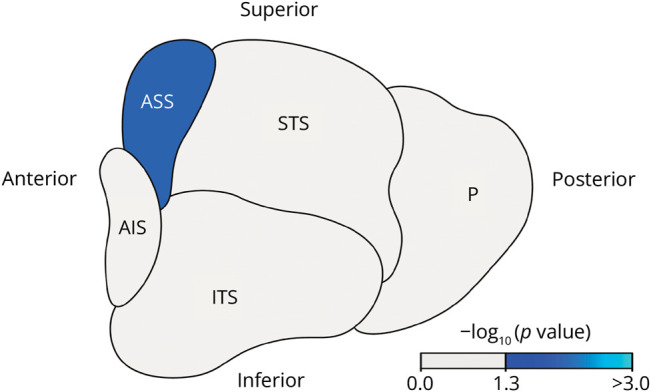
Difference in (Sub)hypothalamic Volumes Between Patients With ALS and Controls Overview of hypothalamic atrophy in patients with ALS compared with controls. Blue regions represent significantly lower volumes in patients with ALS compared with controls after correction for multiple comparisons using permutations. Light gray regions represent no significant difference between groups. The image represents a left lateral view of the averaged left and right hypothalamic volumes. AIS = anterior inferior subregion; ALS = amyotrophic lateral sclerosis; ASS = anterior superior subregion; ITS = inferior tubular subregion; P = posterior subregion; STS = superior tubular subregion.

Differences in hypothalamic volumes between patients with ALS and controls are shown in Figure 1. The volume of the anterior superior subregion was smaller in patients with ALS than in controls (β = −0.70 [−1.15 to −0.25], *p* = 0.013). Total hypothalamic volume and volumes of other subregions did not differ between groups. There were no differences in hypothalamic volumes between *C9orf72*-positive and *C9orf72*-negative patients with ALS, nor between *C9orf72*-positive and *C9orf72*-negative familial controls. Excluding patients with FTD and cognitive or behavioral deficits (sensitivity analysis 1) and patients with weight loss (sensitivity analysis 2) did not change the results.

### Associations With Metabolism

**Figure 2:**
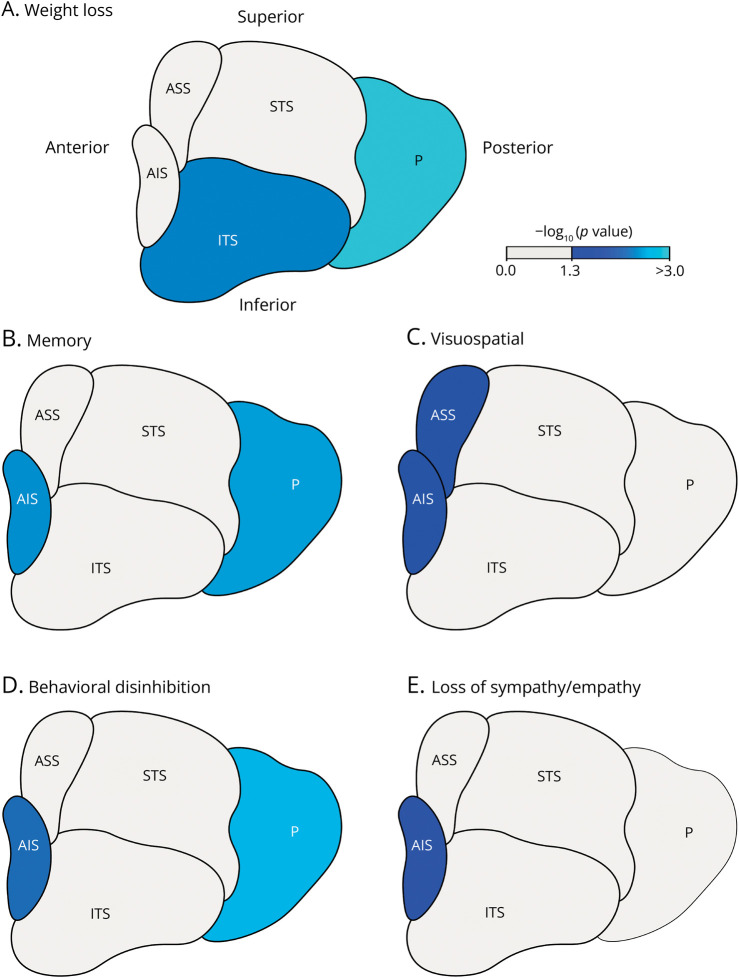
Hypothalamic Correlates of Weight Loss, Cognition, and Behavior Overview of hypothalamic subregions correlated with weight loss at diagnosis (A), cognitive impairments (B, C), and behavioral changes (D, E). In (A), blue regions represent significantly lower volumes of hypothalamic subregions in ALS patients with weight loss compared with those without. Similarly, in (B), this is observed for patients with impaired memory scores, and in (C), for patients with impaired visuospatial scores. These significantly lower volumes (D) in patients with behavioral disinhibition, and (E) in patients experiencing loss of sympathy/empathy. All these differences are significant after correction for multiple comparisons using permutations. Light gray regions represent no significant difference between groups. The image represents a left lateral view of the averaged left and right hypothalamic volumes. AIS = anterior inferior subregion; ALS = amyotrophic lateral sclerosis; ASS = anterior superior subregion; ITS = inferior tubular subregion; P = posterior subregion; STS = superior tubular subregion.

In patients with ALS, weight loss correlated with a smaller volume of the posterior subregion (β = −4.79 [−8.39 to −2.49], *p* = 0.001) and inferior tubular subregion (β = −3.55 [−6.10 to −1.51], *p* = 0.006), as shown in Figure 2A. The difference in total hypothalamic volume (β = −9.00 [−16.53 to −3.37], *p* = 0.016) was driven by volume changes of these 2 subregions. The relationship between hypothalamic volumes and BMI or sex was not affected by ALS. In the subgroup of patients for whom extensive metabolic assessment was available, the posterior hypothalamic volume correlated with both a lower FFM (β = 1.07 [0.14–2.00], *p* = 0.026) and a lower mREE (β = 0.03 [0.003–0.06], *p* = 0.032), as shown in eFigure 1, A and B. There was no correlation between hypothalamic volumes and metabolic index. In the sensitivity analysis, excluding patients with severe atrophy and bulbar symptoms, weight loss still correlated with a smaller posterior volume. The negative correlation between weight loss and inferior tubular subregion volume remained the same but was, due to the reduced power, no longer significant. Finally, there was no significant effect of dysphagia on the relationship between hypothalamic volumes and weight loss.

### Associations With Cognitive Deficits

Impaired memory function was correlated with a smaller volume of the posterior and anterior inferior subregion (β = −10.14 [−15.88 to −4.39], *p* = 0.004 and β = −2.02 [−3.18 to −0.85], *p* = 0.005; Figure 2B). The difference in total hypothalamic volume (β = −17.04 [−29.68 to −4.40], *p* = 0.04) was driven by volume changes of these 2 subregions. Furthermore, abnormal visuospatial scores correlated with a smaller volume of the anterior superior and anterior inferior hypothalamus (β = −1.92 [−3.26 to −0.58], *p* = 0.044 and β = −1.73 [−2.98 to −0.49], *p* = 0.044; Figure 2C). Impaired language, fluency, or executive function did not correlate significantly with (sub)hypothalamic volumes.

### Associations With Behavioral Changes

In patients with ALS, presence of behavioral disinhibition was correlated with a smaller volume of the posterior subregion (β = −12.09 [−18.83 to −5.35], *p* = 0.003; Figure 2D) and anterior inferior subregion (β = −2.17 [−3.54 to −0.81], *p* = 0.010; Figure 2D), which also drove the relationship with a lower total hypothalamic volume (β = −20.83 [−36.17 to −5.49], *p* = 0.036). Loss of sympathy/empathy was correlated with a smaller volume of the anterior inferior subregion (β = −1.62 [−2.80 to −0.43], *p* = 0.034; Figure 2E). In addition, the number of impaired behavioral domains was related to a smaller volume of this subregion (β = −0.55 [−0.95 to −0.15], *p* = 0.031). Presence of apathy, perseverative/stereotyped behavior, or hyperorality/altered eating behavior was not significantly correlated with (sub)hypothalamic volumes. We observed no correlation between hypothalamic volumes and total ALS-FTD-Q score.

### Associations With Clinical Outcomes and Survival

**Figure 3:**
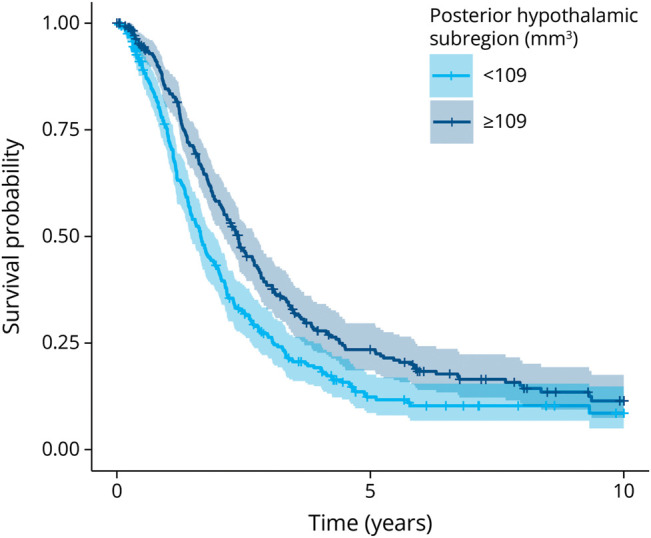
Survival Curve for Patients With ALS Time-to-event analysis according to posterior hypothalamic volume. The plot shows the observed Kaplan-Meier survival curve since first MRI scan, stratified for posterior hypothalamic volume below median (<109 mm^3^) and equal to or higher than median (≥109 mm^3^). Resulting *p* value (*p* = 0.001) was significant after correction for multiple testing using Bonferroni correction. ALS = amyotrophic lateral sclerosis.

The posterior hypothalamus was correlated with shorter survival in the unstratified univariable model (hazard ratio = 0.36 [0.21–0.61], *p* = 0.001, Figure 3). This correlation remained after inclusion of the ENCALS prediction model estimate in the first multivariable model (hazard ratio = 0.49 [0.30–0.87], *p* = 0.011), as well as after correction for weight loss and behavioral changes in the second multivariable model (hazard ratio = 0.33 [0.16–0.68], *p* = 0.003). In the stratified univariable survival analyses, no significant associations between posterior hypothalamic volume and survival were found within the weight loss (n = 177) and behavioral change (n = 95) subgroups. Moreover, no correlation was found between hypothalamic volumes and ALS-FRS-R score or disease progression rate, nor between hypothalamic volumes and symptom duration.

### Longitudinal Assessment of Hypothalamic Volumes

**Figure 4:**
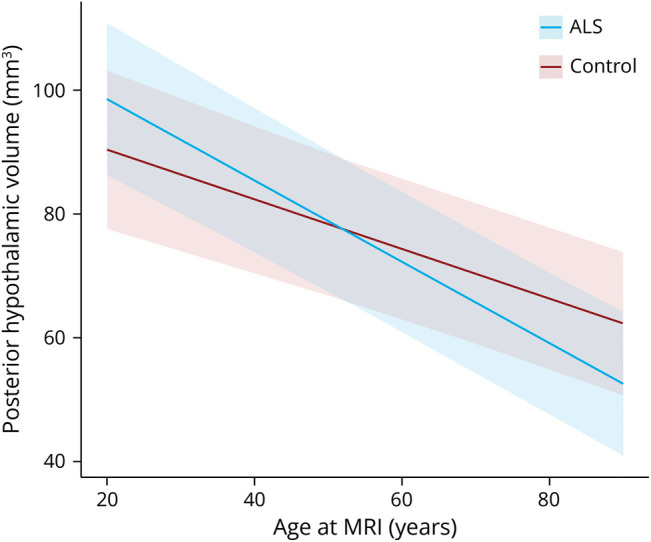
Posterior Hypothalamic Deterioration in Patients With ALS and Controls Interaction analysis to compare posterior hypothalamic change over time between patients with ALS and controls. The resulting *p* value (*p* = 0.029) was significant after correction for multiple comparisons using permutations. ALS = amyotrophic lateral sclerosis.

A total of 628 longitudinal MRI scans were acquired from 313 patients with ALS with a median follow-up duration of 7.43 (5.06–13.57) months; 209 longitudinal MRI scans were obtained from 194 controls with a median follow-up duration of 15.74 (12.67–18.80) months. They were used in addition to the baseline scans, resulting in a total of 1,192 scans of patients with ALS and 565 scans of controls. The volume of the posterior hypothalamic subregion decreased faster over time in patients with ALS than in controls (β = −0.25 [0.42 to −0.09], *p* = 0.029, Figure 4). This did not apply in the other subregions. Finally, weight loss did not affect the deterioration of hypothalamic volumes in patients with ALS.

## Discussion

In this large cross-sectional and longitudinal neuroimaging study covering over 900 participants, we demonstrated that the hypothalamus is involved in ALS. There is selective atrophy in specific subfields, it degenerates faster over time than controls, and it seems to be related to multiple disease characteristics. We were able to investigate previously unknown relationships between the hypothalamus and multiple markers of metabolism, cognition, behavior, and disease progression. We found that the volume of the anterior superior subregion of the hypothalamus was smaller in patients with ALS than in controls, with no overall difference in total hypothalamic volume. Moreover, we found that atrophy of the posterior hypothalamus was associated with weight loss, memory dysfunction, behavioral disinhibition, and survival, and that this subregion deteriorated faster than in controls. These results suggest that the hypothalamus plays a role in metabolic, cognitive, and behavioral disturbances in ALS. This improves our understanding of nonmotor involvement and could have important implications for the identification of new treatment targets for this devastating disease.

Our findings in vivo are in line with earlier postmortem work in 9 patients with ALS that showed selective pathology of the paraventricular nucleus and lateral hypothalamus, which are localized in the anterior superior and posterior subregions.^35^ In contrast to 2 other neuroimaging studies in patients with ALS, we observed no volume loss in the total hypothalamus or the superior tubular subregion.^16,18^ Nor did we find evidence for an effect of *C9orf72* on the hypothalamus of familial controls and patients with ALS, unlike the report in a previous study.^15^ These discrepancies may be due to our larger sample size, automatic (instead of manual) segmentation of the hypothalamus, and the correction for genetic similarity in the comparisons between familial controls in our study. Regarding the association with metabolic disturbances, our investigation yielded no evidence of a relationship with BMI. Although these results differ from earlier publications in ALS, they are consistent with findings in the general population.^15,18,36^ The correlation between weight loss and hypothalamic volume has not been investigated previously. Furthermore, only 1 study correlated hypothalamic volume with metabolic index, comparing normo- and hypermetabolic patients with ALS.^17^ Consistent with our findings, this study found no significant difference in hypothalamic volume between normo- and hypermetabolic patients with ALS.^16^ A recent study of 94 patients with ALS examined the link between hypothalamic volume and cognitive and behavioral changes and, in line with our results, found associations with the anterior and posterior subregions of the hypothalamus. Our findings, however, differed in several aspects. We observed a relationship between hypothalamic volumes and cognitive deficits primarily in the memory and visuospatial domains, but other research found relationships in the memory and fluency domains.^16^ Regarding behavior, we found associations with behavioral disinhibition and loss of sympathy while they found correlations with everyday memory difficulties, reduced motivation, and changes in eating habits. Because the 2 studies used different assessment tools to measure cognitive and behavioral impairments, direct comparisons are limited. Similar patterns of hypothalamic atrophy and correlations with nonmotor symptoms were identified in a study involving 18 patients with behavioral variant FTD, a disease sharing clinical and pathologic overlap with ALS, in comparison with 18 controls.^3,37^ The most pronounced difference was observed in the anterior superior region, consistent with our findings in patients with ALS. In addition, abnormal eating behavior in these patients, often leading to weight changes, was associated with posterior hypothalamus atrophy, aligning with the correlations we observed between posterior hypothalamic atrophy and metabolic and behavioral changes.

Based on our study, we can conclude that atrophy of the anterior superior subregion of the hypothalamus is a disease characteristic of ALS. This was not influenced by disease duration, disease severity, nor the *C9orf72* repeat expansion and could not be detected by comparing the whole hypothalamus between groups. In addition, our longitudinal analysis revealed no deterioration in the anterior superior subregion over time. The paraventricular nucleus, located in the anterior superior subregion, plays an essential role in energy and muscle homeostasis by producing hormones such as oxytocin.^38^ A previous postmortem study showed regional atrophy, TAR DNA-binding protein 43 (TDP-43) inclusions, and a loss of oxytocin-producing neurons in the paraventricular nucleus of patients with ALS.^35^ Perhaps the observed atrophy of the anterior superior subregion is caused by atrophy of the paraventricular nucleus because of degeneration of oxytocin-producing neurons. This might contribute to the development of clinical characteristics such as severe muscle wasting, as seen in ALS.

Atrophy of specific regions within the hypothalamus may contribute to metabolic disturbances, including weight loss, in ALS. Our findings demonstrate that weight loss is associated with reduced inferior tubular and posterior subregion volume. In addition, we found that a lower FFM and decreased mREE are associated with a smaller volume of the posterior subregion. The inferior tubular and posterior subregion include the arcuate nucleus and lateral hypothalamus, 2 nuclei that are part of the melanocortin system. This system regulates food intake and energy expenditure hormonally.^9,10,39^ The lateral hypothalamus produces orexin that has a role in the regulation of eating behavior. Recent findings from a postmortem neuropathologic study in patients with ALS demonstrated TDP-43 inclusions and loss of orexin neurons in the lateral hypothalamus.^35^ This loss of orexin-producing neurons was related to changes in eating behavior.^35^ We hypothesize that atrophy of the lateral hypothalamus, which causes a decreased orexin production, explains the relationship between metabolic alterations (weight loss, lower FFM, and decreased mREE) and a reduced volume of the posterior hypothalamus. Moreover, the antidiabetic drug, pioglitazone, which increases food intake by inhibiting neurons in the melanocortin system, did not result in weight gain among patients with ALS.^39^ This could indicate dysfunction of hypothalamic neurons. Current strategies for preventing or mitigating weight loss focus primarily on high-caloric diets but have limited effect.^40^ If our hypothesis holds true, early intervention may be necessary to prevent weight loss or identify individuals at risk (e.g., those with a smaller posterior hypothalamus and ongoing weight loss).

Our findings demonstrate that hypothalamic involvement in ALS, encompassing both posterior and anterior subregions, is associated with cognitive dysfunction, including memory and visuospatial impairments, and behavioral disturbances such as disinhibition and loss of sympathy. These findings support the notion that the hypothalamus plays an important role in the limbic system. Previous studies in patients with ALS have identified atrophy of limbic structures, such as the hippocampus and amygdala, linked to memory deficits.^41^ Similarly, in FTD, atrophy of limbic structures correlated with disinhibition and impaired emotion recognition.^42^ Despite the established role of limbic structures in memory and behavior, the specific contribution of the hypothalamus to cognition and behavior in ALS remains underexplored. Studies in healthy individuals have demonstrated hypothalamic relationships with various cognitive domains, such as memory processing (including spatial memory and place learning), emotion regulation, and motivated and social behavior.^11,13,43^ Therefore, our results suggest that the hypothalamus, as part of the limbic system, might contribute to the development of memory and visuospatial problems, in addition to behavioral disturbances such as disinhibition and loss of sympathy, in ALS. Moreover, memory deficits are regarded as “nonspecific” cognitive changes in ALS.^44^ Our study showed that abnormalities of the hypothalamus frequently occur in ALS and are related to memory function. This suggests that memory deficits might be more prevalent in ALS than previously acknowledged, although further research is necessary to confirm this hypothesis. In addition, we found a correlation between hypothalamic volumes and behavioral disinhibition, as well as loss of sympathy in ALS, but not with its most common behavioral change, apathy. Apathy has been associated with other brain regions such as the prefrontal cortex, basal ganglia, and parietal regions.^45,46^ However, studies exploring its association with the hypothalamus or investigating brain correlates of behavioral change beyond apathy remain scarce. Our results might suggest that spatially heterogeneous neurodegenerative patterns contribute to various behavioral deficits in ALS, emphasizing the need for further research into their brain correlates.

Our study provides evidence of the vulnerability of the posterior hypothalamus to degeneration in ALS, with posterior hypothalamic atrophy being related to shorter survival and faster deterioration of this subregion over time, compared with controls. Because weight loss is known to be related to shorter survival, and our findings revealed a correlation between weight loss and posterior hypothalamic atrophy,^5^ we investigated whether weight loss in patients affected the posterior hypothalamic deterioration over time. However, we did not observe a significant interaction. In addition, correction for weight loss and behavioral changes in the survival analysis did not change the relationship between posterior hypothalamic volume and survival. In the stratified univariable survival analyses, no significant associations between posterior hypothalamic volume and survival were found within subgroups characterized by weight loss and behavioral changes. This absence of significant findings could potentially be attributed to smaller sample sizes, which may have limited the statistical power of these analyses. Our findings suggest that the effect of posterior hypothalamic atrophy on survival is (partially) independent of weight loss and behavioral change and may indicate distinct underlying mechanisms. The atrophy observed in the posterior hypothalamus could potentially be a consequence of expanding cerebral neurodegeneration, further emphasizing the extensive nonmotor involvement in ALS.

We acknowledge potential limitations to our study. First, the subregions within the hypothalamus are relatively small, making it challenging to segment them accurately. Therefore, we combined left and right subregions for the analyses and, to ensure accuracy, outliers were checked manually. This was done in a blinded manner, ensuring that group and *C9orf72* status did not influence outlier detection. Second, weight loss is associated with various dysmetabolic factors such as dyslipidemia and insulin resistance, which could affect the relationship between weight loss and hypothalamic volumes.^47^ Unfortunately, because of the lack of availability of relevant data, we could not perform this analysis. Furthermore, the relatively small sample size in the subgroup analysis may limit the generalizability of the findings. However, the results are consistent with those of the main analysis. Selection and attrition bias are limitations of all neuroimaging studies in ALS, including ours, because patients whose bulbar or respiratory function is more severely affected are more likely to be excluded, because of their inability to safely remain in a prone position safely. To mitigate this, we included patients as early as possible after diagnosis, supported by similar disease duration of the patients included in this study compared with those not included (eTable 3). A final limitation of this study is the incompleteness of cognitive and behavioral data, primarily because of introduction of the Dutch version of the ECAS in 2015, which limited the availability of detailed cognitive and behavioral data before that date. Although this may introduce some bias, we believe it to be minimal because both the inclusion criteria and MRI scanning procedures remained consistent throughout the study.

Our comprehensive MRI study, encompassing both cross-sectional and longitudinal analyses, provides evidence for hypothalamic involvement in ALS, particularly selective degeneration of the anterior superior subregion. This atrophy could not be detected by comparing the entire hypothalamus between groups and was not driven by the *C9orf72* repeat expansion. Moreover, we demonstrate that posterior hypothalamic atrophy is associated with metabolic dysfunction, memory impairment, behavioral disinhibition, and survival, and, compared with controls, with accelerated degeneration. These findings underscore the role of the hypothalamus in ALS pathogenesis and its relationship with metabolism, cognition, behavior, and survival. Our study contributes to our understanding of the multifaceted nature of ALS and the potential therapeutic implications of targeting hypothalamic dysfunction.

## Appendix Authors

**Table:**

Name	Location	Contribution
Annebelle Michielsen, MD	Department of Neurology, UMC Utrecht Brain Center, University Medical Centre Utrecht, the Netherlands	Drafting/revision of the manuscript for content, including medical writing for content; major role in the acquisition of data; study concept or design; analysis or interpretation of data
Kevin van Veenhuijzen, MD	Department of Neurology, UMC Utrecht Brain Center, University Medical Centre Utrecht, the Netherlands	Drafting/revision of the manuscript for content, including medical writing for content; major role in the acquisition of data; study concept or design
Mark R. Janse van Mantgem, MD	Department of Neurology, UMC Utrecht Brain Center, University Medical Centre Utrecht, the Netherlands	Drafting/revision of the manuscript for content, including medical writing for content; major role in the acquisition of data
Michael A. van Es, MD, PhD	Department of Neurology, UMC Utrecht Brain Center, University Medical Centre Utrecht, the Netherlands	Drafting/revision of the manuscript for content, including medical writing for content; study concept or design
Jan H. Veldink, MD	Department of Neurology, UMC Utrecht Brain Center, University Medical Centre Utrecht, the Netherlands	Drafting/revision of the manuscript for content, including medical writing for content; study concept or design
Ruben P.A. van Eijk, MD, PhD	Department of Neurology, UMC Utrecht Brain Center, and Biostatistics & Research Support, Julius Centre for Health Sciences and Primary Care, University Medical Centre Utrecht, the Netherlands	Drafting/revision of the manuscript for content, including medical writing for content; study concept or design
Leonard H. van den Berg, MD, PhD	Department of Neurology, UMC Utrecht Brain Center, University Medical Centre Utrecht, the Netherlands	Drafting/revision of the manuscript for content, including medical writing for content; study concept or design; analysis or interpretation of data
Henk-Jan Westeneng, MD	Department of Neurology, UMC Utrecht Brain Center, University Medical Centre Utrecht, the Netherlands	Drafting/revision of the manuscript for content, including medical writing for content; major role in the acquisition of data; study concept or design; analysis or interpretation of data

## Glossary

ALS: amyotrophic lateral sclerosis
ALS-FRS-R: revised ALS Functional Rating Scale
ALS-FTD-Q: ALS-FTD Questionnaire
BMI: body mass index
*C9orf72*: chromosome 9 open reading frame 72
ECAS: Edinburgh Cognitive and Behavioural ALS Screen
ENCALS: European Network for the Cure of ALS
eTIV: estimated total intracranial volume
FFM: fat-free mass
FTD: frontotemporal dementia
mREE: measured resting energy expenditure
pREE: predicted resting energy expenditure
TDP-43: TAR DNA-binding protein 43
